# Motif Enrichment Tool

**DOI:** 10.1093/nar/gku456

**Published:** 2014-05-23

**Authors:** Charles Blatti, Saurabh Sinha

**Affiliations:** 1 Department of Computer Science, University of Illinois at Urbana-Champaign, Urbana, IL 61801, USA; 2 Institute for Genomic Biology, University of Illinois at Urbana-Champaign, Urbana, IL 61801, USA

## Abstract

The Motif Enrichment Tool (MET) provides an online interface that enables users to find major transcriptional regulators of their gene sets of interest. MET searches the appropriate regulatory region around each gene and identifies which transcription factor DNA-binding specificities (motifs) are statistically overrepresented. Motif enrichment analysis is currently available for many metazoan species including human, mouse, fruit fly, planaria and flowering plants. MET also leverages high-throughput experimental data such as ChIP-seq and DNase-seq from ENCODE and ModENCODE to identify the regulatory targets of a transcription factor with greater precision. The results from MET are produced in real time and are linked to a genome browser for easy follow-up analysis. Use of the web tool is free and open to all, and there is no login requirement. Address: http://veda.cs.uiuc.edu/MET/.

## INTRODUCTION

Researchers frequently design and conduct experiments that result in a novel set of functionally related genes. It is a common paradigm of genomic analysis to understand these experimentally derived gene sets by statistically associating them with previously characterized sets from literature and public databases. An example of this approach (1) identifies the Gene Ontology (2) terms that are enriched in a novel gene set obtained from co-expression analysis. There are numerous popular web services, such as the Database for Annotation, Visualization and Integrated Discovery (DAVID) (3), that are designed to identify these statistical associations. In fact, a 2008 survey (4) catalogued 30 separate web tools dedicated to this important task. These tools differ in their approaches to identify meaningful associations and by their collections of curated gene sets.

**Figure 1.**
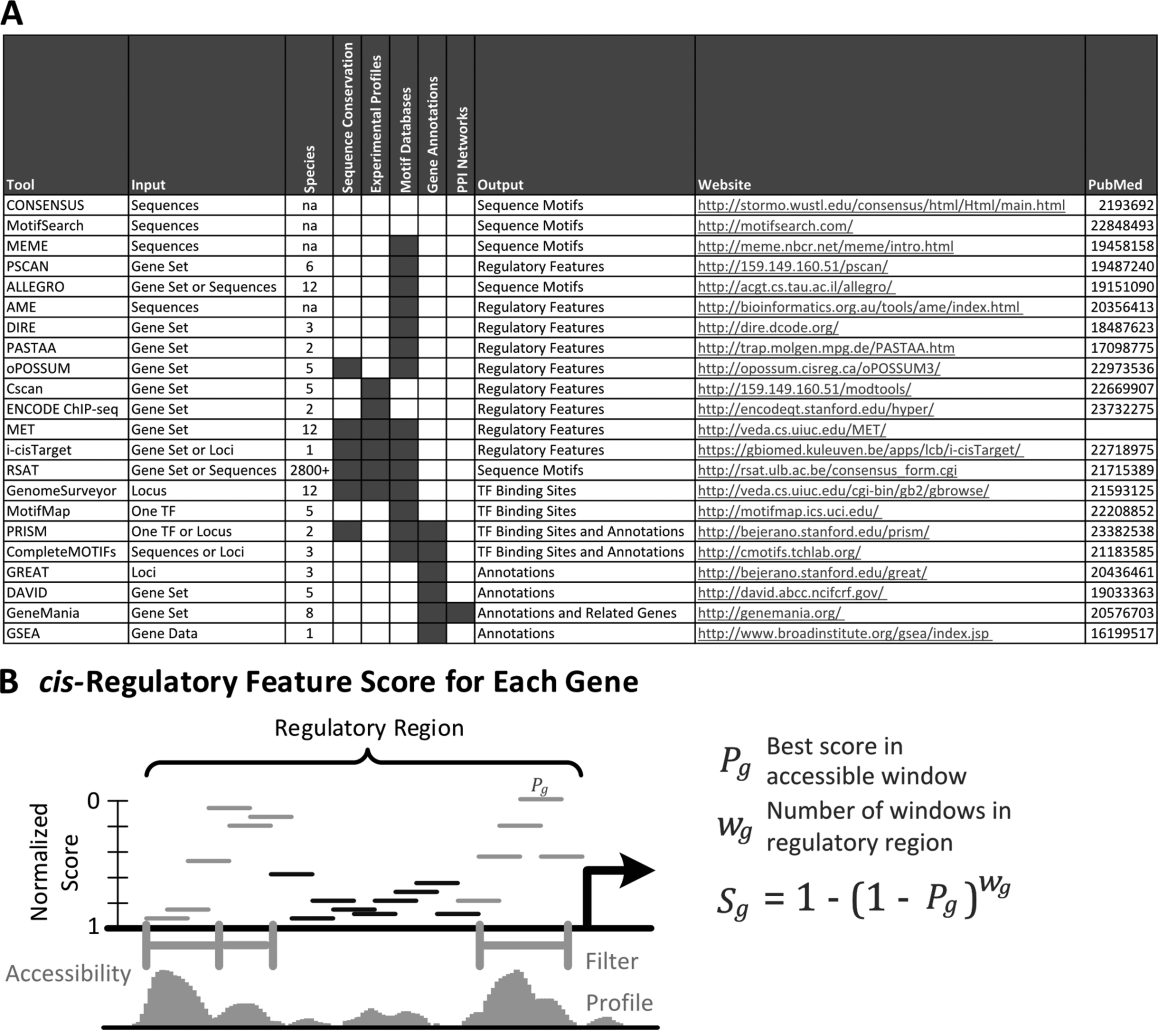
(**A**) Comparison of online tools for gene set and *cis*-regulatory analysis. Each tool is listed along with its typical input and expected output. The grayed cells indicate the types of data sources used by each tool in its analysis. The website URLs and PubMed ID references are also provided. (**B**) Visualization of calculating a regulatory feature score for a gene. Shown is a particular regulatory region of a gene. The horizontal bars within the regulatory region represent the normalized feature score of each window as calculated by MET for a single TF motif with higher bars representing better scores. Below in gray is a chromatin accessibility profile of the genomic locus and the thick gray bars indicate accessible regions where MET will consider the motif-based scores. The regulatory feature score of the gene for this example TF motif is calculated as shown on the right using the best score in the accessible windows of the regulatory region.

Fewer tools exist that take the genes of an experimentally-derived set and examine their corresponding non-coding regions for evidence of a shared regulatory signature. This is an important analysis that can uncover major transcriptional regulators of the novel gene set and suggest a mechanistic explanation for the results of the experiment. The most common type of analysis is to subject the regulatory sequences of the novel gene set to *ab initio* motif-finding tools, such as Multiple EM for Motif Elicitation (MEME) (5). These tools identify short, over-represented deoxyribonucleic acid (DNA) patterns that may then potentially be mapped to known transcription factor motifs. One disadvantage of this type of approach is that it searches over the large space of all possible motifs, which may result in the loss of statistical power. Motif-scoring tools, e.g. PRISM (6), take collections of experimentally characterized motifs and search for their occurrence in each genomic loci provided by the user. The new web-based tool presented here, called ‘Motif Enrichment Tool’ (MET), extends this motif scoring approach and attempts to predict the major regulators of the provided gene set by testing if the non-coding sequences of its genes are enriched in the motifs from experimentally determined collections. MET quantifies the presence of a motif with a probabilistic score that integrates both weak and strong binding sites embedded in a genomic segment rather than simply counting the number of sites that are strong matches to the motif. MET offers users the option of using chromatin accessibility profiles (DNase-seq data), if available, to improve functional binding prediction. The web tool also provides the option of refining its computational predictions of transcription factor binding locations based on sequence conservation across multiple species. In addition to identifying motif over-representation, MET can discover common regulators of a gene set that are revealed by TF-DNA binding profiles from chromatin immunoprecipitation (ChIP) experiments. The methods underlying MET have been used in previous publications on songbirds (7), honeybees (8), other insects (9) and in studies on human, stickleback fish and mouse. The i-cisTarget tool (10) is similar to MET in its goals and capabilities, although it uses different data collections and methods to calculate conservation and enrichment. i-cisTarget covers the fruit fly genome, while MET analysis is currently available for a dozen species with more species to be added soon. Figure 1A compares a number of related online analysis tools for novel gene sets by their expected input, the public-domain data they incorporate and the results they return.

## MATERIALS AND METHODS

The MET performs enrichment tests on user-defined gene sets to identify their potential major transcriptional regulators. It does so by defining a set of genes that are putative regulatory targets of a TF and quantifying the significance of its overlap with the user gene set. The procedure for defining regulatory target genes has several steps, many of which can be customized by the user.

First, a genome-wide scoring profile of a TF's binding (henceforth called a ‘*cis*-regulatory feature’) is created either computationally from motifs or from ChIP data. This profile assigns a score to every 500 bp window in the genome (in shifts of 250 bp), representing the strength of that feature (e.g. motif presence or ChIP occupancy) in the window. MET currently supports ChIP-based analysis for 143 ChIP data sets from lymphoblastoid cell lines in humans and 53 ChIP data sets of early embryonic development in fruit fly (11). A genome-wide ChIP profile is pre-processed to produce a single value representing the average ChIP signal (e.g. read counts) for each genomic window.

Since ChIP data is not available for most species, MET also scores each genomic window by computationally predicting TF binding from its DNA sequence and characterized TF-DNA binding specificities (motifs). MET uses the HMM-based program Stubb (12) to compute a score that integrates over both strong and weak matches to the motif present in a window. MET scores TF motifs from several database collections. It utilizes vertebrate motifs from JASPAR (13) and TRANSFAC (14), which catalog footprinted binding sites from literature. MET also scores a collection of 239 non-redundant motifs of human TFs that were characterized using the high throughput SELEX method (15). For analysis of insect genomes, MET supports a collection of 223 non-redundant motifs from the FlyFactorSurvey database (16) that were characterized with a bacterial one-hybrid system. 108 motifs of *Arabidopsis* TFs characterized with protein binding microarrays (17) provide the basis for MET analysis in plants. MET allows the user to select the collection of regulatory features (e.g. motif database or ChIP data source) they wish to examine for associations with their gene set.

The next step in the MET pipeline is to rank-normalize the regulatory feature profiles, converting the original feature values (motif scores or ChIP scores) into scores from 0 to 1 where 0 represents the best value. For instance, a window that scores in the top 1% genome-wide would be given a normalized score of 0.01. A variant of this normalization procedure considers the local G/C content. The motivation is straightforward. If a motif is composed of mostly C's and G's, then a high Stubb score is expected to be computed in a G/C rich window. We are interested in those windows where the motif matches are much stronger than expected by G/C content alone. Thus, the ‘G/C normalization’ procedure separates genomic windows into 20 equal-sized bins based on their G/C content, and performs rank-normalization within each bin separately. MET allows the user to choose between standard normalization and G/C normalization.

Motif scoring is a noisy process with a high level of false positives. To make our method more accurate, we incorporate sequence conservation and chromatin accessibility data where available. For *Drosophila melanogaster*, there are 11 other available *Drosophila* genomes. To score a *D. melanogaster* window, we combine the Stubb scores of the 12 orthologous windows in a phylogenetic-weighted averaging scheme (18), causing windows with a conserved motif presence to be scored higher. These multi-species averages are then subjected to the rank-normalization procedure. Another method for increasing the accuracy of predicted TF-DNA binding profiles is by using cell type specific chromatin accessibility data as a filter. Currently, DNaseI hypersensitivity (DHS) profiles from different stages of fruit fly early embryonic development (19) are available for use as optional filters. Windows that score in the top 10% for accessibility in a given stage are retained for further analysis, and all other windows are assigned a rank-normalized score of 1 (no binding). MET also supports an accessibility filter for human genome analysis based on DHS data from lymphoblastoid cell lines generated by the ENCODE project (20). Where available, MET allows the user to select particular accessibility filters and/or multi-species based motif scores to increase the specificity of the genome-wide motif profiles to be examined.

Once the *cis*-regulatory feature profile has been defined and normalized, MET identifies the genes that have the strongest feature scores in their regulatory regions. This is called the ‘target gene set’ of the motif or TF that defines the feature. In this step, the user is allowed to choose how to define the regulatory regions of genes. The user may choose regions of a predefined, fixed length, e.g. ‘1 kbp upstream’, ‘10 kbp upstream’, or ‘5 kbp upstream and 2 kbp downstream’ of the transcription start site (TSS). MET also offers two additional types of regulatory regions of variable length: ‘nearest TSS’ defines regulatory regions as the genomic windows that are closer to the gene's TSS than to any other TSS, while ‘gene territory’ includes the gene's body and half the distance to the nearest non-overlapping genes upstream and downstream. Once a regulatory region type has been selected, MET produces a score, *S_g_* , for each gene *g* for the presence of a given *cis*-regulatory feature in that gene's regulatory region. This is given by:

\begin{equation*} S_g = 1 - (1 - P_g )^{w_g} \end{equation*}

where *P_g_* is the best normalized score of the regulatory feature in the regulatory region of *g* and *w_g_* is the number of windows in the region. To create the target gene set corresponding to the feature (motif or TF), MET selects a fixed number of genes with the best scores. Figure 1B shows the normalized score profile for a single motif in the regulatory region of gene filtered by chromatin accessibility and the components for computing the corresponding regulatory feature score.

Ultimately, MET tests the significance of overlap between the target gene set of each regulatory feature and the user-provided gene set using a one-sided Fisher's exact test. It displays a list of significantly enriched features ranked by the *P*-values of these tests, and also provides links for understanding and investigating the reported statistical associations. MET can quickly produce these results for multiple user sets and many possible combinations of user options, since most of the necessary information is pre-computed and only the last step of the significance test with the user-provided gene sets is performed in real time. Simultaneously examining the results of many different methodological options allows the user to discover the most informative normalization methods, window filters and regulatory region types.

## WEB SERVER

### Configuration pages

#### Navigation page

The main navigation page of the Motif Enrichment Tool provides a description of the tool as well as links to related resources. Most importantly, this page requires the user to select their species of interest. MET currently supports 12 species ranging from vertebrates (human, mouse, zebra finch, stickleback) to insects (fruit fly, honeybee, red flour beetle, parasitoid wasp, yellowfever and African malaria mosquitos) to planaria and to flowering plants (arabidopsis). Additional species, motif collections and high throughput experimental profiles are added regularly.

#### Options configuration page

**Figure 2.**
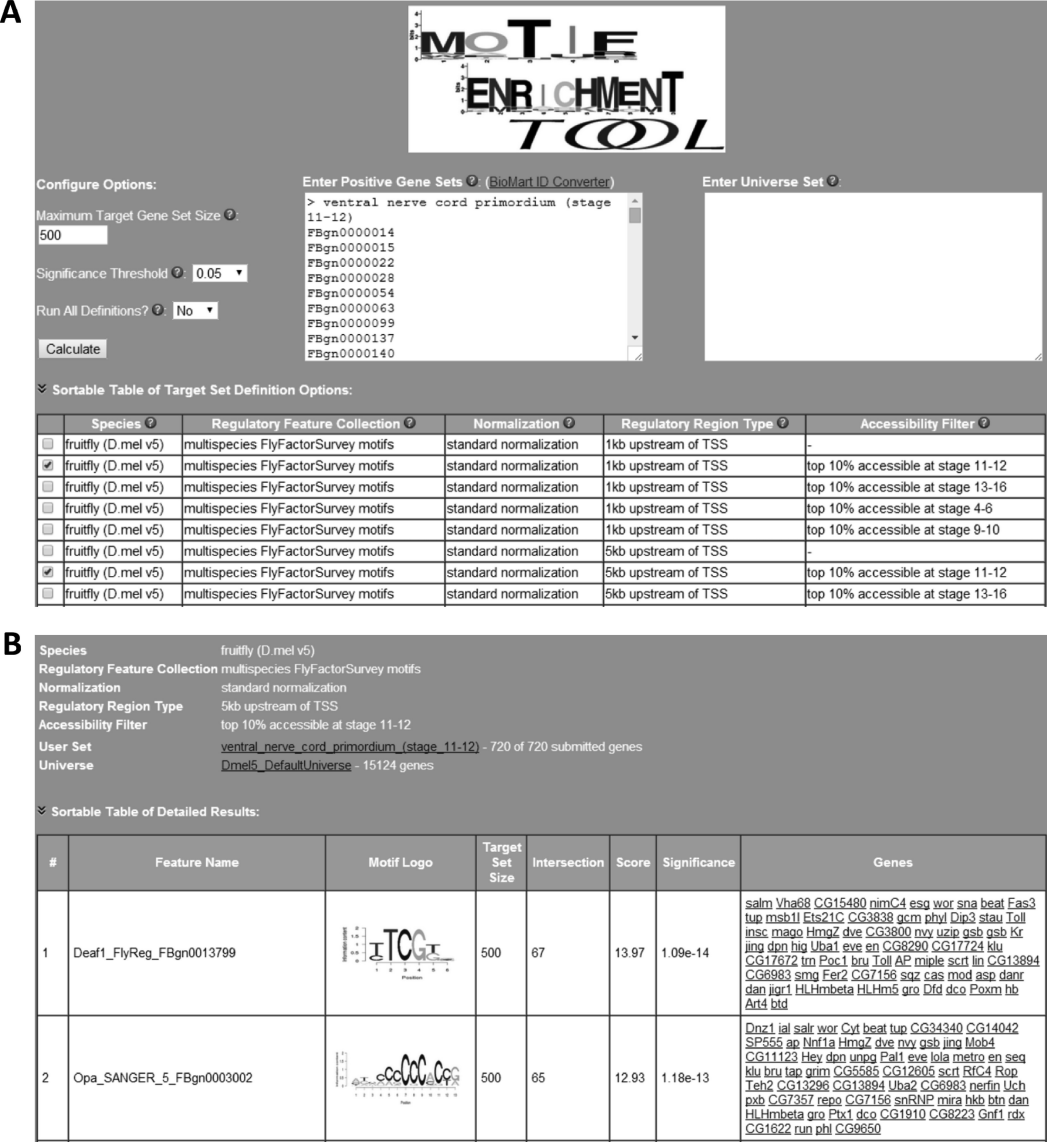
(**A**) Screenshot of options configuration page. In this example, the user has entered their gene set of interest, the set of genes that are expressed in the ventral nerve cord primordium of stages 11–12 *Drosophila* embryos. No gene universe has been entered, so the default universe for *Drosophila* will be used. The user has selected to perform the enrichment tests with two different ways to define the motif target gene sets. Because the gene set was defined in stages 11–12, they have selected options that use chromatin accessibility data from the matching time point. The difference in their two selections is in the size of the defined regulatory region. (**B**) Screenshot of the results details page. The results are from the second configuration option the user selected in panel A. At the top are the details of the configuration and links to lists of the gene sets involved. The table shows the top two enriched regulatory features for this nerve cord gene set. The most enriched feature (significance 10e−14) is the motif for the transcription factor DEAF1 and its DNA-binding specificity logo is shown. The 67 nerve cord genes that are likely targets of DEAF1 are listed in the rightmost column. Each gene name provides a link to a genome browser view of its regulatory region where the motif scoring profile can be visualized.

Upon choosing a species, the user is directed to the options configuration page as illustrated by Figure 2A. The first step is to enter their gene set of interest in the central panel. An example set is preloaded in the text box, and shows the user the correct format and the type of gene identifiers that must be used for that particular species. There is a link to the BioMart ID Converter (21) to help the user find the correct gene IDs. The MET tool supports multiple user gene sets to be tested simultaneously if entered in the correct format in this text box. In the rightmost text box, the user may enter a universe of genes to consider for the analysis. By default, MET defines the gene universe as all of the genes in its gene annotation file for that species. The user may want to narrow down the gene universe if there were genes excluded from their experimental analysis, e.g. a microarray experiment with no probes for some genes. The user may set the maximum size of the target gene sets (500 by default) and the minimum *P*-value significance to display associations. Each row in the table at the bottom of the page represents the regulatory target gene sets corresponding to a particular collection of features (motif database or ChIP data source), with a particular normalization method, regulatory region type and accessibility filter. The user may select one, many, or all (using the ‘Run All Definitions’) of these definitions of target gene sets to perform enrichment tests. We included a detailed example analysis on our web page to demonstrate how a user might consider the results of multiple definitions.

### Results pages

#### Summary page

The results summary page loads progressively as each batch of enrichment tests is performed on the user's gene set(s). Results are saved on the server for up to 2 weeks and can be accessed using the address linked to at the top of the page. Each row in the sortable summary table represents one group of enrichment tests between a user gene set and target gene sets defined by a particular combination of options. There are additional columns to indicate how many and what fraction of those enrichment tests were significant, as well as a column that lists the names of the most significantly enriched regulatory features. The first column provides links to detailed results for further inspection by the user. A text file that contains the results from all enrichment tests is provided below the table to facilitate additional manipulation of the results in a spreadsheet.

#### Detailed results page

The top of a detailed results page shows the combination of options used to define target gene sets. Sizes and elements of the user-provided gene set and the gene universe are also available at the top of this page. The enrichment tests are reported below in a sortable table. When the regulatory feature is a TF motif, a visual representation (logo) of the DNA binding specificity is provided. This is followed by the size of the target gene set, its overlap with the user gene set and the significance of the enrichment test (as well as the negative log_10_ of the *P*-value). The final column lists the genes that were found in the overlap, i.e. those user-provided genes that are also targets of the regulatory feature. Each gene name is a link to its regulatory region in a genome browser called Genome Surveyor (22) which specializes in the display of experimental and computationally predicated regulatory features. With additional configuration in Genome Surveyor, the significantly enriched regulatory features can be displayed as a track for deeper analysis of the result. Figure 2B shows the detailed results of a sample query in which the user searched for potential regulators of genes expressed in the ventral nerve cord primordium of stages 11–12 fruit fly embryos (23). The top TF motif discovered in the 5 kbp upstream regulatory regions while using sequence conservation and stage-specific chromatin accessibility filter, was DEAF1, a transcription factor that has been identified as a regulator of neural tube development in mouse embryos (24).

## CONCLUSION

The novel Motif Enrichment Tool fulfills an important role in biologists’ enrichment analysis pipeline of experimental gene sets by identifying potential transcriptional regulators. Since MET uses computational scans of TF motifs, it currently operates on a dozen genomes and can be extended to any species with available genome sequence and annotation. MET has mechanisms for incorporating high throughput data and sequence conservation to improve regulatory feature prediction. Because of the variability of genome and regulatory sequence characteristics, MET allows a user to quickly explore the different methods for defining regulatory feature target sets and choose the most appropriate for their analysis.
